# Differential gene expression of the honey bee *Apis mellifera *associated with *Varroa destructor *infection

**DOI:** 10.1186/1471-2164-9-301

**Published:** 2008-06-25

**Authors:** M Navajas, A Migeon, C Alaux, ML Martin-Magniette, GE Robinson, JD Evans, S Cros-Arteil, D Crauser, Y Le Conte

**Affiliations:** 1 INRA, UMR CBGP (INRA/IRD/Cirad/Montpellier SupAgro), Campus International de Baillarguet, CS 30016, F-34988 Montferrier-sur-Lez Cedex, France; 2 Institute for Genomic Biology, Department of Entomology, University of Illinois at Urbana-Champaign, 505 S. Goodwin Ave., Urbana, IL 61801, USA; 3 INRA, UMR AgroParisTech/INRA MIA 518, 16 rue Claude Bernard, F-75231 Paris Cedex05, France; 4 INRA, UMR INRA/1165/CNRS 8114/UEVE Unité de Recherche en Génomique Végétale, 2 rue Gaston Crémieux, F-91057 Evry Cedex, France; 5 USDA-ARS Bee Research Laboratory, BARC-E Bldg 476, Beltsville, MD 20705, USA; 6 INRA, UMR 406 Ecologie des Invertébrés, Site Agroparc, 84914 Avignon Cedex 9, France

## Abstract

**Background:**

The parasitic mite, *Varroa destructor*, is the most serious pest of the western honey bee, *Apis mellifera*, and has caused the death of millions of colonies worldwide. This mite reproduces in brood cells and parasitizes immature and adult bees. We investigated whether *Varroa *infestation induces changes in *Apis mellifera *gene expression, and whether there are genotypic differences that affect gene expression relevant to the bee's tolerance, as first steps toward unravelling mechanisms of host response and differences in susceptibility to *Varroa *parasitism.

**Results:**

We explored the transcriptional response to mite parasitism in two genetic stocks of *A. mellifera *which differ in susceptibility to *Varroa*, comparing parasitized and non-parasitized full-sister pupae from both stocks. Bee expression profiles were analyzed using microarrays derived from honey bee ESTs whose annotation has recently been enhanced by results from the honey bee genome sequence. We measured differences in gene expression in two colonies of *Varroa*-susceptible and two colonies of *Varroa*-tolerant bees. We identified a set of 148 genes with significantly different patterns of expression: 32 varied with the presence of *Varroa*, 116 varied with bee genotype, and 2 with both. *Varroa *parasitism caused changes in the expression of genes related to embryonic development, cell metabolism and immunity. Bees tolerant to *Varroa *were mainly characterized by differences in the expression of genes regulating neuronal development, neuronal sensitivity and olfaction. Differences in olfaction and sensitivity to stimuli are two parameters that could, at least in part, account for bee tolerance to *Varroa*; differences in olfaction may be related to increased grooming and hygienic behavior, important behaviors known to be involved in *Varroa *tolerance.

**Conclusion:**

These results suggest that differences in behavior, rather than in the immune system, underlie *Varroa *tolerance in honey bees, and give an indication of the specific physiological changes found in parasitized bees. They provide a first step toward better understanding molecular pathways involved in this important host-parasite relationship.

## Background

The honey bee (*Apis mellifera*, Insecta: Hymenoptera) has become an important model for genetic study, especially as its genome has been sequenced [1]. It is also an important economic insect as it is the world's principal crop pollinator and honey producer [2]. These activities have been threatened by the spread of *Varroa destructor *(Acari: Parasitiformes) a parasite of honey bees that causes devastating harm in many countries [3]. *Varroa *mites are ectoparasites of honey bees, parasitizing immature and adult bees and reproducing in cells in the honeycomb that contain brood [4]. *Varroa *mites or virus associated to mites impair the honey bee immune system [5] and in some cases boost the amplification of bee viruses [6]. The mechanisms underlying the mite's suppression of bee immunity and its impacts on pathogen virulence have not been elucidated.

There are genetic differences in the ability of honey bees to tolerate *Varroa *parasitism (tolerance is defined here as the capacity for the bees to survive when the parasite develops, preventing the demise of the hive). Some colonies of honey bees tolerate *Varroa *infection and survive despite the presence of the parasite in the hive [7-10]. These differences have been attributed to a variety of factors, including grooming and hygienic behavior [4], and differences in the timing of larval and pupal development that impact mite reproduction [11].

Colonies of the Asian honey bee *Apis cerana *(the original host of *V. destructor*[12]) suffer less damage from this parasite than *A. mellifera *in spite of the presence of the mite in the hives, and these two factors have been implicated [13]. The mechanisms underlying genetic differences for honey bee tolerance to *Varroa *mites are unknown. Insights into these mechanisms may lead to new molecular tools for both *Varroa *diagnosis and selective breeding of mite-tolerant honey bees for the bee industry. These issues are now amenable to study thanks to new genomic resources available for honey bees. Microarray analyses of differences in gene expression due to both mite parasitization and genotypic differences in bee tolerance are powerful approaches to explore the role of many genes in what are no doubt multifactorial resistance traits. Microarrays have proven to be useful in the study of host-pathogen interactions in other insects [14-18].

## Results

### Differences in bee gene expression in response to Varroa infestation and bee genotype

Comparisons of *Varroa*-infected and non-infected pupae from the 4 colonies studied pinpointed the expression of 32 genes whose expression varied significantly between the two types of pupae. Among them, 15 (47%) of these cDNAs were significantly up-regulated and 17 (53%) down-regulated in bees exposed to *Varroa*. The magnitude of the difference of expression is small in all cases. The single exception was EST (BB160020A20G03) which was over-expressed 20-fold in bees infected with *Varroa*. BLAST searches indicated that this EST matches the honey bee virus (AY292384), deformed wing virus [19].

There were 116 cDNAs whose expression varied with bee genotype, 47 (40.5%) of them up-regulated and 69 (59.5%) down-regulated. Two genes were regulated both as a function of *Varroa *infection and bee genotype. *Dlic *2 is down-regulated in *Varroa*-parasitized bees and up-regulated in tolerant bees, whereas *Strn-Mlck *is down-regulated both in *Varroa*-parasitized and tolerant bees. The full list of genes with significant differences in expression is presented in Additional file 1.

### Identification of *A. mellifera*-regulated transcripts and assignment of biological functions

To add biological meaning to the relatively large amount of microarray-derived data, we searched the GeneOntology database for the putative biological processes and molecular functions for the genes that showed significant differences in expression. Among these 148 genes, 19 associated with the presence of *Varroa *and 68 associated with bee genotype had information in Gene Ontology.

To unravel functional differences related to *Varroa *parasitism and bee genotype, we analyzed sets of genes for overrepresentation in functional GO categories according to biological process and molecular function (Additional file 2). Regarding biological process, genes involved in the regulation of protein metabolism, embryonic development and reproduction show a significant enrichment among the categories tested for the genes that varied in expression with *Varroa *infestation. A set of genes involved in cytokinesis, nervous system development, behavior, signal transduction and transcription-DNA dependent emerged from the analysis of genes that varied with bee genotype. The cluster analysis showing genes sharing the same GO term is shown in Table 1. Most of the genes affected by *Varroa *infection are involved in protein metabolism and their function is related to transferase and catalytic activity. The set of genes differentially expressed between tolerant and sensitive bees are mainly involved in transcription and neuron development. These genes have a nucleic acid binding function and are associated with pyrophosphatase GTPase, transferase and catalytic activity.

**Table 1:** Functional clustering based on Gene Ontology

**(A) Varroa**			
	*P *value	Up-regulated	Down-regulated

**Biological process **(unknown)		6 (9)	10 (6)
cell organization and biogenesis	0.000229	2	3
protein metabolism	0.004808	4	5
cellular metabolism	0.008242	4	6
protein modification	0.035714	2	1
**Molecular function **(unknown)		5 (9)	9 (8)
transferase activity	0.021978	3	0
catalytic activity	0.041958	3	3

**(B) Bee phenotype**			

	*P *value	Up-regulated	Down-regulated

**Biological process **(unknown)		24 (20)	24 (31)
regulation of physiological process	3.279e-08	3	6
regulation of transcription	2.650e-09	3	5
Axonogenesis	5.139e-06	0	4
cell division	0.000231	0	3
Localization	1.450e-05	7	2
axon guidance	5.782e-05	0	3
Phosphorylation	7.709e-05	1	3
cell organization and biogenesis	0.000155	5	2
nervous system development	7.359e-05	1	4
protein modification	0.000360	3	1
**Molecular function **(unknown)		20 (24)	26 (29)
ATPase activity	5.428e-05	2	1
nucleic acid binding	3.480e-07	4	5
methyltransferase activity	5.428e-05	1	2
pyrophosphatase activity	4.720e-06	3	1
ion transporter activity	5.428e-05	2	1
ubiquitin-protein ligase activity	5.428e-05	2	1
kinase activity	0.001086	2	1
catalytic activity	2.904e-06	6	8
transmembrane receptor protein kinase activity	5.428e-05	0	3
cytoskeletal protein binding	0.000543	0	3
transferase activity	1.339e-09	3	7
ion binding	1.101e-05	2	2
signal transducer activity	0.002336	0	4

Functional clustering based on Gene Ontology of genes that show significant differences are indicated for bees parasitized by Varroa (A), and for Varroa-tolerant bees (B). Gene Ontology terms are non-exclusive. Expression patterns within these sets of genes are represented (number of genes that are significantly up- or down-regulated). Genes with unknown biological process or molecular function are in brackets.

### RT-qPCR analysis

**Figure 1:**
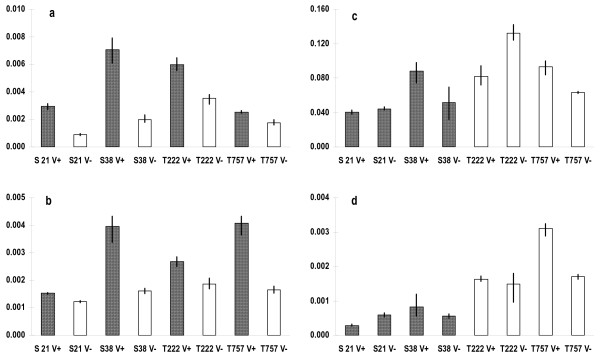
**cDNA levels of four transcripts shown by microarray analysis to be significantly regulated in *Apis mellifera***. In a and b, samples of bees infested with *Varroa *(hatched bars) are compared with bees free of *Varroa *(white bars). In c and d: *Varroa*-sensitive bees (hatched bars) are compared with *Varroa*-tolerant bees (white bars). All the values shown are mean ± SE.

To further evaluate our ability to detect significant variation in gene expression at low fold-difference levels, a set of 4 functionally annotated bee transcripts was subjected to RT-qPCR analysis (Table 2). Differently expressed levels of cDNAs were quantified and are shown in Fig. 1. In accord with microarray analysis, cDNAs from *Varroa*-parasitized bees were up regulated for genes *baz *and CG9520 (unassigned) (Fig. 1a and 1b); or from tolerant bees for genes *Alh *and *Hr78 *(Fig. 1c and 1d). In all cases, expression levels quantified by qPCR are represented by the mean of 3 measurements (maximum and minimum values are indicated in Fig. 1).

**Table 2:** Characteristics of the RT-qPCR analysis of differentially expressed genes of *Apis mellifera*

Product name	Honey bee ID	Forward primer sequences	Reverse primer sequence	Amplicon size (bp)	Amplification efficiency^a^(R^b^)
*Krh1*	GB14867	ACTCATCAGTTGTTGGTTCTCCTC	TCGTTTGGCTCTTCAGTCTTGTG	118	2.11 (0.99)
non determined	GB18554	TCACACCGATATTCTCATCAAAGG	CTTGTCATTCTTGTTCTCCGATTG	112	1.94 (0.98)
*Hr78*	GB18358	TGACGAAGTTTAGTTGCTGCTATG	TGTTGTTCCCTATGATCTCTGTCC	107	1.98 (0.99)
*Alh*	GB17400	ACTTGTGGTAATGCTGGCTGAC	AACGAACGAAGGAAAGGAATAACG	129	2.08 (0.96)
*baz*	GB10346	ACCAGGAACAAGCGAGTCAGAAG	ACCAGGAACAAGCGAGTCAGAAG	112	2.02 (0.99)

Honey bee IDs refer to the cDNAs selected from the EST microarray experiment (see text).

*Krh1 *is the housekeeping gene. Forward and reverse primer sequences and the PCR product length are indicated.

^a^Amplification efficiencies were calculated from the slope of standard curves as E = 10 [-1/slope] – 1.100% efficiency corresponds to an amplification efficiency of 1; ^b^Regression coefficient of linear standard curve.

## Discussion

Previous gene expression studies concerning honey bee immunity have mainly investigated responses to microbial pathogens [20]. Yet physiological responses to macroparasites such as *Varroa *are likely to be very different from microorganisms, as has been shown recently in a study on *Drosophila *revealing the ability of this species to activate a systemic immune response adapted to the invader [16]. The results described here pinpoint several genes regulated by *Varroa *parasitism that can be linked to honey bee responses to the presence of *Varroa *or to differences in bee tolerance.

### Honey bee biological response to *Varroa *parasitism

**Figure 2:**
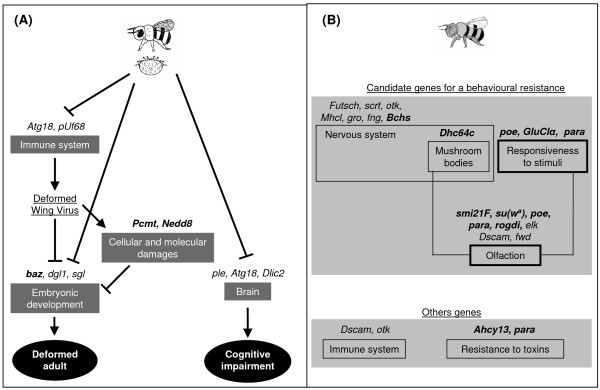
**Hypothetical pathways and models of honey bee responses to *Varroa*-parasitism (A) and the bee tolerant genotype (B)**. Arrows and dashes indicate positive and negative regulation, respectively. Gene names in bold are up-regulated. In A) one of the consequences of the *Varroa *parasitism is a decline in immune capacity which induces the proliferation in bees of the Deformed Wing Virus (DWV). The boost of DWV multiplication might cause cellular and molecular damage, inducing the production of protein repair (*Pcmt*) and the labelling of proteins for degradation (*Nedd8*). In addition, regulated genes that might be affected by the presence of the DWV are indicated. Mites might decrease the production of dopamine (*ple*) and inhibit genes known for indirectly preventing neural degeneration in aged adults, which could explain the cognitive impairment often observed in adults parasitized by *Varroa*. In B) different genes can be associated to behavioral tolerance to *Varroa*. Tolerant and non-tolerant bees differ significantly by the expression of genes involved in the nervous system development. The olfactory pathway and neurons excitability seem also to play an important role in *Varroa*-tolerance. See text for a full discussion on the genes involved in the pathways presented.

The pathways suggested by this study to be associated with honey bee response to *Varroa *parasitism are schematized hypothetically in Fig. 2. Responses to *Varroa *can be grouped in two main categories, one related to deformed bee adults occasioned by the presence of the Deformed Wing Virus (DWV) often associated with *Varroa *parasitism, and a second category of genes that are related to cognitive impairment.

### *Varroa *parasitism and deformed wing virus

One of the consequences of *Varroa *parasitism is a decline in immune capacity which appears to induce the proliferation of viruses such as deformed wings virus in bees [6]. The down-regulation of the autophagic-specific gene 18 (*Atg18*) detected here is noticeable. Autophagy is an important mechanism for innate immunity against bacteria and viruses [21,22]. The candidate innate immunity gene *poly U binding factor 68 Kd *(pUf68) [23] is also down-regulated in *Varroa *parasitized bees. By decreasing autophagy and immunity processes in bees, *Varroa *might favor the proliferation of DWV. Interestingly, the *Varroa*-parasitized bees displayed high levels of DWV viral RNA (about 20-fold; Additional file 1). The boost of DWV multiplication might cause cellular and molecular damage, and thus the observed production of genes for protein repair (*Pcmt) *[24], and the labeling of proteins for degradation (*Nedd8*) [25]. In contrast to these findings, we did not see a decrease on transcript abundance of immune pathway members [20] (Evans et al., 2006) found on this array. In fact, transcripts for the gene *Rab7*, a plausible regulator of immunity, were up-regulated in *Varroa*-parasitized bees.

The most notable symptoms of *Varroa*-parasitized bees are disfigured, small adults with deformed legs and wings [4,6]. Our results show that at the transcriptome level the presence of *Varroa *down-regulates two genes involved in developmental processes, *slg *and *dlg1*. It has been shown that the *Drosophila sugarless *(*sgl*) gene regulates *wingless *signaling [26], which has a critical role in developmental processes. The gene *dlg1 *has been implicated in the control of proliferation of *Drosophila *imaginal discs programmed to produce adult structures at metamorphosis [27]. Although a down-regulation of these genes could have been induced by the presence of *Varroa *(via the DWV virus), a specific link with the development of deformed adults of virus-infected bees would need to be further investigated.

### Cognitive impairment in bees parasitized by *Varroa*

*Varroa *infestation does not always cause wing deformity. Adults may sometimes appear to be normal morphologically, but there are mite effects on adult bee behavior. In particular, mite-parasitized foragers display a decrease in learning capability [28], prolonged absences from the nest and a lower rate of return to the colony [29]. A decrease in neuronal capacities involved in learning and navigation is a possible cause. Although the physiological mechanisms underlying reduced performance by bees in the presence of *Varroa *remain unknown, our results show that, the gene *pale *encoding tyramine hydroxylase is down-regulated in pupae parasitized by *Varroa*. Interestingly, the gene *pale *is needed for dopamine synthesis, which stimulates the nervous system and has many functions in the brain, including important roles in neural development, behavior and cognition, motor activity, motivation and learning. It is also interesting to note that the *Dlic2 *and *Atg18 *genes, both down-regulated in *Varroa*-parasitized bees, are enhancers of the *blue cheese *gene (*bchs*)[30], which is up-regulated in tolerant bees (see below). The *bchs *gene has been reported as preventing progressive neural degeneration in aged flies [31]. It is plausible, if these changes are chronic (still expressed in adults), that infested bees have a higher rate of neuronal apoptosis when aging, which might explain why foragers (the oldest bees in the nest) have difficulties in learning and orientating in flight.

### Behavioral resistance of bees to *Varroa*

Grooming behavior seems to be one of the main characters involved in the tolerance of bees to *Varroa *[4]. Similarly, selection for tolerance to *Varroa *seems to be possible by selecting bees with a high level of hygienic behavior [32]. Hygienic behavior is understood as the ability of bees to uncap and remove infected brood. Although the molecular mechanisms underlying this trait remain unknown, some insights are provided here based on the large-scale comparative analysis of transcriptome differences between *Varroa*-tolerant and sensitive bees.

A disproportionately high fraction of the genes differentially expressed between tolerant and susceptible bees are involved in the development of the nervous system (Fig. 2B). A large part of these genes are down-regulated in tolerant bees compared to sensitive bees, including the genes *futsch, scratch (scrt), otk, Myosin heavy-chain-like (Mhcl), groucho *(*gro), kekkon*-1 (*kek1*) [26,33-38]. *Fringe *(*fng*), also down regulated in *Varroa*-tolerant bees, plays an important role in the positive regulation of Notch signalling pathway involved in the regulation of genes that control multiple cell differentiation processes during embryonic and adult life, such as neuronal function and development [39]. Finally, a gene involved in locomotory behavior, *single-minded *(*sim*), is down-regulated in tolerant bees. *Drosophila *mutant *sim *flies are only able to walk in circles and this phenotype is due to defects in the central brain complex [40].

Several genes involved in neuron excitability are up-regulated in *Varroa*-tolerant bees compared to sensitive bees. *Purity of essence *(*poe*) (also named *pushover*) is involved in neuronal excitability and may play a role in responsiveness to environmental stimuli and behavior. For example, fly mutants display behavioral defects such as sluggishness, uncoordination and defective flight [41]. This gene has been shown to be down-regulated in honey bees by the queen mandibular pheromone, which slows the behavioral maturation of workers, e.g. transition from inside hive work (nurse) to foraging activity(forager) [42]. The *GluClα *gene, a glutamate-gated Cl^- ^channel specific to arthropods, which is also up-expressed in tolerant bees, is known to modulate neuronal membrane excitability [43]. The paralytic gene (*para*), known to be important in the conducting of nerve action potentials in flies [44], is also up-regulated. Seen together, these genes suggest a mechanism by which *Varroa*-tolerant bees are more sensitive to external stimuli than *Varroa *sensitive bees. Also, the Dynein heavy chain 64C gene (*Dhc64c*), up-regulated in tolerant bees, is required for proliferation of mushroom-body neuroblasts [45]. Mushroom bodies have an important role in insect cognition and are known to be involved in learning and memory, particularly for smell.

Interestingly, several genes involved in olfaction (smell impaired 21F *smi21F*, suppressor of the white-apricot gene *su(w*^*a*^*)*, *poe*, *para, Rogdi*) are up-regulated in *Varroa*-tolerant bees compared to sensitive bees [46-50]. However, the Down syndrome cell adhesion molecule gene (*Dscam*) and *four wheel drive *(*fwd*) also have a role in olfaction, and they are down-regulated in *Varroa*-tolerant bees [34]. The differential expression of this group of genes depending on bee genotype is of major importance considering that in the hive, olfaction and neuronal sensitivity together may play a major role in the detection of *Varroa *infested cells. Observations made on two earlier generations of the bee stocks here studied have been shown that *Varroa*-tolerant bees have better ability to detect the mite [51]. Previous studies showed that hygienic bees have a higher olfactory sensitivity and responsiveness than non-hygienic bees [52-54]. They are notably able to discriminate between odors of healthy and diseased brood at a lower stimulus level, suggesting that olfaction and responsiveness play a key role in hygienic behavior. In addition, observations made on the behavior of the brood, have shown open cells containing destroyed parasitized bee pupae in tolerant experimental colonies, which is in agreement with a bee hygienic behavior against *Varroa*. If these gene candidate pathways for *Varroa *behavioral tolerance can be further confirmed, the results suggest that they already set up during the pupae stage.

There were differences in expression between tolerant and sensitive bees for genes involved in increased resistance to toxins like Ahcy13 (*Adenosylhomocysteinase at 13*) involved in detoxification [55], and *para *involved in resistance to insecticide pyrethroids and DDT (Dichloro-Diphenyl-Trichloroethane) [56]. Two genes linked to immunity, *Dsam *and *otk*, members of the Immunoglobulin gene superfamily in *Drosophila *[57] were down-regulated in *Varroa*-tolerant bees, while one galectin-family gene (an apparent ortholog to Drosophila CG32226) appears to be upregulated.

### Low fold differences in gene expression

For most of the genes showing significant differences in expression in the present study, the magnitude of these differences was small. Recent reports have shown that microarrays can significantly underestimate gene expression changes and therefore if a severe cut-off is applied, this approach might miss important changes in gene expression. Recent reports have validated the capability of a microarray approach to detect small gene expression differences [58]; 87% of a set of parasite-specific genes displayed changes 2-fold or less in *D. melanogaster *challenged to a protozoan parasite [17]. Similarly, about 60% of genes of the lepidopteran, *Spodoptera frugiperda*, revealed around 0.5-fold changes in the transcript levels associated with virus infection [14]. As one possible explanation for low level gene regulation, it has been suggested in Drosophila that much of the response to parasite attack probably does not involve de novo gene expression but post-transcriptional events [18]. Another possibility is that many small differences in gene expression reflect subtle modulation by a large number of factors acting in cascade. The small changes reported here might also represent an underestimation of tissue-specific effects obscured by whole-body analysis. Future studies of *Varroa *effects on honey bees should analyze specific tissues, and our results suggest a focus on the brain would be fruitful. Previous studies have demonstrated extensive regulation of brain gene expression in conjunction with honey bee behaviour [42,59-61].

## Conclusion

This work is the first step towards understanding the genomic responses of honey bees to *Varroa *parasitism. It demonstrates that honey bee pupae exhibit differences in gene expression associated either with the presence of *Varroa*, or with tolerance to this parasite. These results highlight the potential importance of behavioral mechanisms of response to *Varroa *and suggest that a study focused on the brain is of importance for the future. For an economically important species such as the honey bee, the identification of parasite-specific response factors might ultimately serve to identify molecules that act on bee parasites. In addition, differences between tolerant and sensitive bees could lead to developing tools to select improved strains of honey bees for beekeepers.

## Methods

### Honey bee colonies and sample collection

Both, sensitive and tolerant honey bee colonies here studied, belong to non related local strains of the same *A. mellifera *population which is bred in the Laboratory of Bee Biology and Protection, Institut National de la Recherche Agronomique, Avignon, France. Four colonies were used for this study; two (T222 and T757) had survived for 11 years with no chemical treatment against *Varroa *despite the widespread presence of this parasite in the locale, and displayed a very low rate of parasitism [10]. The other two colonies (S21 and S38) showed a very high level of parasitism. No acaricide treatment was applied to avoid pesticide bias. Parasitism intensity was determined by counting the number of mites that died naturally and accumulated on the floor of the hive in each colony during the year (method described in Ellis et.al. [62]). Estimations based on data for 5 years (4 measurements/year) showed that the infestation rate in colonies S21 and S38 was on average 10 times higher than in colonies T222 and T757.

The reproduction of *Varroa *mites can be affected by traits in developing bees [63,64]. We therefore examined gene expression in pupae. Honey bee pupae were collected from the four bee colonies at the blue-eye stage at the start of cuticle pigmentation, making it possible to determine the bee developmental stage [65]. Capped brood cells were opened and two samples of 50 parasitized and non-parasitized pupae were collected. A pupa was considered to be parasitized if a reproductive *Varroa *was found with it in the honeycomb cell. The pupae were collected and snap frozen using N2 and stored at -80°C until RNA extraction.

We compared parasitized and non-parasitized pupae from both susceptible and tolerant colonies. To minimize the effect of genetic variation, pools of full-sister pupae (related by 75% due to haplodiploidy [66]) were collected in equal numbers from each of the study colonies.

### Sibling estimation by microsatellite genotyping

To identify full sisters, one hundred bees per colony were genotyped (50 parasitized and 50 not parasitized) using *Ap53 *and *A107 *microsatellite loci; these loci have been shown to be effective for this purpose [67]. DNA was extracted from 2 legs/pupa, and DNA extraction and PCR amplification were as described [67], except that the PCR products were detected on a MegaBACE DNA Analysis System 1000 (Molecular Dynamics Inc., USA). The genotype data were used to assemble sets of 8 pupae belonging to the same full sibling group. For each of these, subsets of pupae of *Varroa*-parasitized and non parasitized bees were analyzed in microarray experiments. This procedure minimizes the effects of intra-colonial genetic variation on gene expression.

### Microarray experimental design

**Figure 3:**
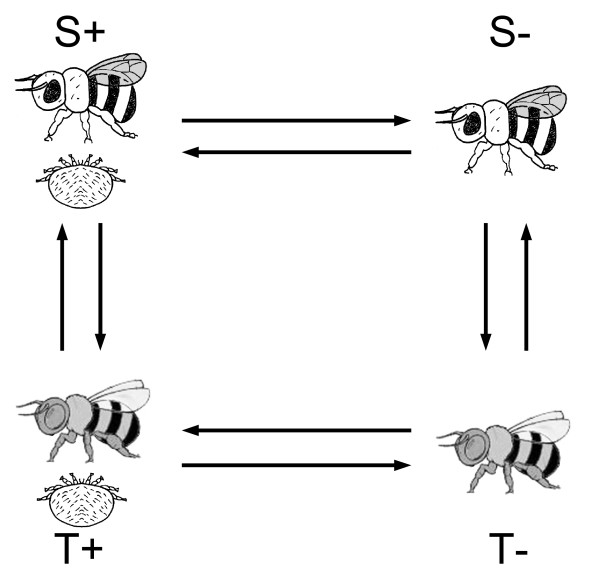
**Microarray experimental design**. For all experiments, arrows indicate microarray hybridizations (arrow tail, Cy3-labeled sample; arrowhead, Cy5-labeled sample). *Varroa *parasitized (+) and non-parasitized (-) full-sister pupae, from two different genetic stocks: one susceptible (S) and one tolerant (T) to *Varroa *were compared. Dye swaps were made for all comparisons.

We compared parasitized and non-parasitized pupae from both susceptible and tolerant colonies using a direct loop design. Direct comparisons were made between parasitized and unparasitized bees from sensitive colonies and from tolerant colonies (Fig. 3). All comparisons were performed in dye-swap on 2 biological samples. A total of 8 samples were thus contrasted using 16 arrays in all (2 biological replicates and 2 technical replicates). Each sample consisted of a fraction of the total RNA obtained from a pool of 8 bees. Full-sister bees were assembled as pools from specific patrilines detected in each colony.

### RNA isolation

The sets of bees were freeze-dried and ground in liquid nitrogen. 30 mg (about 3% of the powder obtained) was used for each RNA extraction using the RNeasy Plant Mini Kit (Qiagen, Courtaboeuf, France) following the manufacturer's protocol for animal tissues. The total RNA solution was treated in liquid by RNase-Free DNase I and purified on column RNA Cleanup (Qiagen, Courtaboeuf, France) according to the manufacturer's protocols. The purity and concentration of RNA were determined by OD measurements in a spectrophotometer. RNA extraction was validated by specific PCR amplifications following RT-PCR of both the glyceraldehyde 3 phosphate dehydrogenase 1 (XM_397363) and the epsilon-tubulin 1 (XM_394700) genes. The quality of the extracted RNA (integrity and size distribution of total RNA) was verified by 1.2% agarose gel electrophoresis in TAE buffer and ethidium bromide staining.

### cDNA synthesis and array hybridizations

10 μg total RNA was used for cDNA synthesis using fluorescent Cy3-dCTP or Cy5-dCTP (Amersham Biosciences, Orsay, France) and the Pronto!TM plus System kit (Promega, Charbonnières, France) for labeling. Dye-labeled cDNAs were purified on cleanup columns (Promega, Charbonnières, France). 55 pmol of the labeled cDNA per dye was used per slide. Prehybridation, hybridization and washing steps were performed according to [61]. The slides were hybridized at 42°C and air dried by centrifugation for 2 min at 800 rpm at room temperature.

### Microarray data acquisition and statistical analysis

The arrays were scanned on a GenePix 4000A scanner (Axon Instruments, Foster City, USA) and images were analyzed by GenePix Pro 3.0 (Axon Instruments, Foster City, USA). For each array, the raw data comprised the logarithm (base 2) of median feature pixel intensity at wavelength 635 nm (red) and 532 nm (green). No background was subtracted. We excluded control spots (as described in [61]) and spots for which duplicates did not pass quality controls standards, cDNAs were not found by spot-finding software, or those determined to be irregular by visual inspection. Intensity signals for cDNAs passing these filters were normalized for intensity- and position-dependent bias. Array-by-array normalization was performed to remove systematic biases. Then, we replaced the value of the spots that were considered as badly formed features with the value of the duplicate. We averaged the two values from each duplicated feature to obtain one value of the gene per array in each condition.

A total of 4,795 cDNAs passed initial filters; 3,045 of these were collapsed to genes and 1,750 ESTs were unassigned. We refer to the combined set of genes and unassigned ESTs as genes, although some redundancy might exist. ANOVA analysis was used to classify the differentially expressed genes according with the different factors considered (P < 0.05 was used to denote statistical significance).

Statistical analyses were conducted using R version 2.2.0 [61] and the R/maanova software package version 0.98.8 [61,68]. The gene-by-gene mixed effect ANOVA model, Yijkvr = μ + Ai + Dj + (ST)k + Vv + Eijkvr, was applied. Observation Yijkvr is the expression value of the gene in the biological replicate r studied on array i when the condition is labelled with dye j. The condition is defined by a modality of the genotype, say k, and the modality of the *Varroa*, say v. The residual of the mode Eijkvr and the term Ai are treated as random effects and the others as fixed effects. Statistical tests were performed with R/Maanova using the hybrid variance model, Fs, based on a James-Stein estimator [69,70]. To control the number of false-positives, the significance (nominal *P*-value) of genes was computed using a permutation test (pvalperm with 1000 permutations) [70]. Permutation tests have the advantage of not assuming a parametric underlying distribution of expression values. A gene was declared differentially expressed if its adjusted *P*-value is lower than 0.05. The adjusted *P*-value used here made it possible to control the Family Wise Error Rate (FWER)

### Functional analysis of gene expression

Microarray ESTs were annotated as described in [60,71]. Briefly, ESTs corresponding to microarray cDNAs were tested for near-perfect matches (98% identity) to coding (protein) sequence or to genomic sequence within or immediately downstream (500 bp) of predicted genes (using release 1 of the honey bee Official Gene Set [72]). Redundant cDNA values were averaged (by using untransformedvalues), and resulting values were assigned to official gene names (which are all prefixed "GB"). Remaining cDNAs not associated with predicted sequences retained their EST identifiers and are presented here by EST accession number (prefixed "BB"). Genes were tentatively assigned molecular function terms based on annotation of the single best BLASTX match in *Drosophila melanogaster*.

We used Gene Ontology (GO) analysis to identify the biological function of the differentially expressed genes, as described in [60]. We sought statistically overrepresented terms among the set of genes significantly regulated. This Enrichment of Gene Ontology (GO) category was tested with a Hypergeometric test followed by the Benjamini Hochberg correction for multiple testing using the analysis tool GOToolBox [60]. Briefly, the analysis calculates the frequency of terms of a gene list (the significantly regulated genes) and compares these results with total frequencies of genes analyzed. We also grouped functionally related genes on the basis of their GO terms. Distances based on GO terms are calculated for all possible pairs from the gene list which are then used for clustering (e.g. the probability that they are functionally related). The functional clustering of genes was carried out in GOToolBox using the WPGMA algorithm. A minimum cluster size of 3 genes was applied.

### Transcript quantification by RT-qPCR

The results of the microarray study were confirmed by measuring the expression of 4 differentially regulated genes (Table 2) using Real-Time quantitative PCR (RT-qPCR). Using an aliquot of the RNA extractions (pools of 8 bees) used for the microarray work, cDNAs were created using the ImProm-II™ Reverse Transcription System kit (Promega). This cDNA (three replicates) was used as template for the Real-Time PCR performed in a Roche LightCycler 480 Instrument platform. The reaction mix consisted of 10 μl of LightCycler 480 SYBR Green Supermix (Roche Laboratories), 5 μl (either 600 nM or 300 nM) of forward and reverse primers, 3 μl dH_2_O and 2 μl cDNA template. Three reactions were performed and means calculated for each locus and treatment. To standardize the results, a housekeeping gene (*Rp49) *that did not vary in expression was used as control. The forward/reverse primer sequences are indicated in Table 2. The efficiency for each locus was determined by running a dilution series (1000x, 100x, 10x, 1x) in triplicate. The results were standardized using the [60] method. Efficiency of the amplicons obtained for each locus was adequately high and at least 97% (Table 2).

## Authors' contributions

MN participated in conceiving the project and in designing the experimental setup, oversaw the research and primarily wrote the paper. AM participated in the planning of all experiments and performed statistical analysis. CA developed behavioral interpretation of results and actively contributed to the writing of the paper. MLM–M designed statistical analysis. GER provided support to apply honeybee microarray resources and contributed to discussion of results. JDE performed analysis of results related to immunity and contributed to discussion of overall results. SC–A performed technical work and contributed to data acquisition. DC performed technical beekeeping experiments. YLC participated in the conception and design of the study, conceived and carried out all biological experiences and participated in discussion of overall results. All authors read, edited, and approved the final manuscript.

## Supplementary Material

Additional file 1 **List of *Apis mellifera *genes with significantly different expression profiles in Varroa-parasitized and non-parasitized bees (A); in two types of bee genotypes (either sensitive or tolerant to Varroa) (B)**. The EST ID, the official honey bee gene ID, the gene name of the best symbol of *Drosophila melanogaster *(based on Flybase information) and the fold change in gene expression, are indicated for both up-regulated and down-regulated transcripts. Unassigned genes are indicated by *. Click here for file

Additional file 2 **Enrichment analysis on genes that show significant differences in bees parasitized by Varroa (A) and between two different bee genotypes (tolerant and sensitive to Varroa) (B)**. GO ID and GO term refer respectively to Gene Ontology ID and the associated term. Click here for file
