# Supplementary material for: Differential gene expression of the honey bee Apis mellifera associated with Varroa destructor infection
Source: BMC Genomics. 2008 Jun 25;9:301. doi: 10.1186/1471-2164-9-301 (PMC2447852; doi:10.1186/1471-2164-9-301)
Supplement: Additional file 1 — List of Apis mellifera genes with significantly different expression profiles in Varroa-parasitized and non-parasitized bees (A); in two types of bee genotypes (either sensitive or tolerant to Varroa) (B). The EST ID, the official honey bee gene ID, the gene name of the best symbol of Drosophila melanogaster (based on Flybase information) and the fold change in gene expression, are indicated for both up-regulated and down-regulated transcripts. Unassigned genes are indicated by *. [file 1471-2164-9-301-S1.doc]

### Additional file 1 – List of *Apis mellifera* genes with significantly different expression profiles in Varroa-parasitized and non-parasitized bees (A); in two types of bee genotypes (either sensitive or tolerant to Varroa) (B).

The EST ID (http://www.life.uiuc.edu/robinson/research/est.html), the official honey bee gene ID, the gene name of the best symbol of Drosophila melanogaster (based on Flybase information: http://flybase.bio.indiana.edu/) and the fold change in gene expression, are indicated for both up-regulated and down-regulated transcripts. Unassigned genes are indicated by *.

A) Varroa-parasitized and non-parasitized bees

| **Varroa up regulated** | | | |  | **Varroa down regulated** | | | |
| --- | --- | --- | --- | --- | --- | --- | --- | --- |
| **EST ID** | **Honey bee ID** | **Best Fly Symbol** | **Fold change (+/-)** |  | **EST ID** | **Honey bee ID** | **Best Fly Symbol** | **Fold change (+/-)** |
| BB160007B20F04 | GB14680 | *CG14082* | 1.49 |  | BB160007B10D05 | GB16058 | *dlg1* | 0.93 |
| BB160007A20C12 | * | * | 1.09 |  | BB160012B10H01 | GB13651 | *pUf68* | 0.93 |
| BB160019B20C03 | GB10882 | *Pcmt* | 1.10 |  | BB160014A20G12 | * | *** | 0.91 |
| BB160020A20G03 | * | *** | 20.32 |  | BB160014B10F07 | GB16686 | *** | 0.78 |
| BB160021B20D09 | * | *** | 1.07 |  | BB160020B20A07 | GB18191 | *RpS23* | 0.81 |
| BB160024B20H03 | GB19424 | *CG11180* | 1.10 |  | BB160020B20B06 | * | *** | 0.83 |
| BB170007B20C07 | GB15772 | *Rab7* | 1.09 |  | BB160020B20D04 | GB17303 | *Atg18* | 0.94 |
| BB170008B10F03 | * | *** | 1.05 |  | BB170001A20H09 | GB16748 | *Dlic2* | 0.85 |
| BB170008B20G12 | GB17785 | *Nedd8* | 1.04 |  | BB170002A10F05 | GB18810 | *CG5337* | 0.90 |
| BB170020A10A01 | GB18554 | *CG9520* | 1.15 |  | BB170002B20H02 | GB14290 | *Strn*Mlck* | 0.93 |
| BB170022B10H08 | * | *** | 1.09 |  | BB170007A20E09 | GB15000 | *sgl* | 0.87 |
| BB170023B10C06 | GB12891 | *CG11859* | 1.09 |  | BB170008A10C04 | GB12868 | *** | 0.92 |
| BB170025B10B10 | * | *** | 1.08 |  | BB170017B10E03 | GB18265 | *** | 0.86 |
| BB170026A10G10 | * | *** | 1.09 |  | BB170025B10C01 | GB15303 | *ple* | 0.70 |
| BB170031A20H10 | GB10346 | *baz* | 1.13 |  | BB170026B20F09 | GB10515 | *CG9338* | 0.87 |
|  |  |  |  |  | BB170030B20B04 | * | *** | 0.91 |
|  |  |  |  |  | BB170030B20C03 | GB10028 | *CG8974* | 0.89 |

B) Sensitive or tolerant to Varroa bees genotypes

| **Tolerant up regulated** | | | |  | **Tolerant down regulated** | | | |
| --- | --- | --- | --- | --- | --- | --- | --- | --- |
| **EST ID** | **Honey bee ID** | **Best Fly Symbol** | **Fold change (T/S)** |  | **EST ID** | **Honey bee ID** | **Best Fly Symbol** | **Fold change (T/S)** |
| BB160010B20D04 | * | * | 1.16 |  | BB160003A10C03 | GB17604 | *fng* | 0.84 |
| BB160011A20B12 | GB14901 | *CG17996* | 1.11 |  | BB160003A20F10 | GB10218 | *CG7991* | 0.84 |
| BB160012B20G06 | GB19272 | TBPH | 1.31 |  | BB160003A20H11 | * | * | 0.84 |
| BB160013B10B01 | * | *** | 1.26 |  | BB160003B10B10 | GB11336 | *CG8979* | 0.84 |
| BB160016B20A12 | * | *** | 1.16 |  | BB160003B10D12 | * | *** | 0.79 |
| BB160016B20D09 | GB11325 | *su(w^a^)* | 1.11 |  | BB160004A10D05 | GB12236 | *** | 0.48 |
| BB160016B20F06 | GB10249 | *bchs* | 1.14 |  | BB160004A10E06 | GB16653 | *** | 0.86 |
| BB160017A10A03 | GB11687 | *Pi3K59F* | 1.14 |  | BB160004A10E11 | GB12249 | elk | 0.85 |
| BB160017A20D04 | * | *** | 1.16 |  | BB160004A10G05 | * | *** | 0.83 |
| BB160020B10A03 | GB18324 | *CG32226* | 1.19 |  | BB160004A20A12 | GB13456 | *gro* | 0.84 |
| BB160020B20A03 | GB18993 | *smi21F* | 1.15 |  | BB160004A20E11 | * | *** | 0.82 |
| BB160020B20C08 | GB12929 | *para* | 1.11 |  | BB160005A10H05 | GB17490 | *kek1* | 0.85 |
| BB160021B20C02 | * | *** | 1.20 |  | BB160005A10H10 | * | *** | 0.85 |
| BB170001B10G11 | GB10711 | *Ucp4A* | 1.30 |  | BB160005A10H11 | * | *** | 0.82 |
| BB170002A20G10 | GB17400 | *Alh* | 1.10 |  | BB160005A20D10 | GB13772 | *Psa* | 0.93 |
| BB170003A10G04 | GB16392 | *CG11414* | 1.13 |  | BB160005A20G10 | GB14838 | *RpIII128* | 0.87 |
| BB170004A10B08 | GB13043 | *CG15440* | 1.08 |  | BB160005A20H03 | * | *** | 0.84 |
| BB170004A20E11 | * | *** | 1.12 |  | BB160005A20H05 | GB10180 | *scrt* | 0.81 |
| BB170009A10E04 | GB11056 | *Pgk* | 1.13 |  | BB160005B10D12 | GB19870 | *fwd* | 0.90 |
| BB170011B10B11 | GB10654 | *Dhc64C* | 1.09 |  | BB160005B20A12 | GB16075 | *CG13211* | 0.76 |
| BB170013A10A03 | GB16748 | *Dlic2* | 1.10 |  | BB160005B20F05 | GB17327 | *CG4300* | 0.84 |
| BB170013A10F11 | * | *** | 1.14 |  | BB160005B20F05 | GB17327 | *CG4300* | 0.84 |
| BB170013A20A08 | * | *** | 1.20 |  | BB160006A10F07 | * | *** | 0.83 |
| BB170013B10H06 | GB16240 | *GluCl** | 1.14 |  | BB160006A10F09 | GB19322 | *CG12802* | 0.82 |
| BB170014A20F03 | * | *** | 1.12 |  | BB160006A10H05 | GB17458 | *Pnn* | 0.85 |
| BB170014A20G10 | * | *** | 1.10 |  | BB160006A20D06 | GB18602 | *CG7582* | 0.88 |
| BB170015B10D04 | GB18358 | *Hr78* | 1.16 |  | BB160006A20G02 | GB14773 | *CG32663* | 0.90 |
| BB170016A10B02 | GB18198 | *Karyβ3* | 1.17 |  | BB160006A20G04 | GB16845 | *Mhcl* | 0.88 |
| BB170018A10C10 | GB19493 | *G*oα47A* | 1.19 |  | BB160006B10C09 | GB16625 | *CG32169* | 0.83 |
| BB170019A10F02 | GB14324 | *Ahcy13* | 1.15 |  | BB160006B10D02 | GB18511 | *CG3996* | 0.80 |
| BB170019B10H02 | * | *** | 1.15 |  | BB160007A10A10 | GB18974 | *** | 0.85 |
| BB170019B20C11 | GB11375 | *** | 1.27 |  | BB160007A10E10 | GB15141 | Dscam | 0.85 |
| BB170020A10A08 | * | *** | 1.10 |  | BB160007A10F07 | GB12369 | *dpr9* | 0.83 |
| BB170021A10C09 | * | *** | 1.12 |  | BB160007A10H01 | GB12914 | *CG9339* | 0.88 |
| BB170021B20G11 | GB12850 | *CG10171* | 1.12 |  | BB160007A20C03 | GB19385 | *Suv4*20* | 0.87 |
| BB170027B20C10 | GB10608 | *Mo25* | 1.05 |  | BB160007A20C12 | * | * | 0.90 |
| BB170027B20G04 | GB16772 | *** | 1.17 |  | BB160007A20F08 | * | *** | 0.85 |
| BB170028A20A03 | GB10967 | *Pcmt* | 1.13 |  | BB160007B10C03 | * | *** | 0.86 |
| BB170028B10B03 | * | *** | 1.08 |  | BB160007B10F08 | GB12499 | *CG1815* | 0.83 |
| BB170028B10D03 | GB12151 | *CG14998* | 1.09 |  | BB160007B20B05 | GB19433 | *CG9399* | 0.86 |
| BB170029A20D06 | GB10659 | *CG10186* | 1.15 |  | BB160008A10B08 | GB11453 | *CG11063* | 0.90 |
| BB170030A10A02 | GB13111 | *rogdi* | 1.21 |  | BB160008A10D01 | * | *** | 0.84 |
| BB170030B20H12 | GB10276 | *CG8408* | 1.17 |  | BB160008A20E12 | * | *** | 0.84 |
| BB170031A10D04 | * | *** | 1.15 |  | BB160008A20G09 | GB15356 | *syd* | 0.89 |
| BB170031B10F12 | GB12737 | *Sirt4* | 1.16 |  | BB160008B10G02 | GB14030 | *CG16728* | 0.88 |
| BB170032A20C07 | GB15671 | *poe* | 1.12 |  | BB160008B20C02 | * | *** | 0.85 |
| Hsp60xxxxxxxxx | * | *** | 1.31 |  | BB160008B20F03 | * | *** | 0.90 |
|  |  |  |  |  | BB160009A20B03 | GB13152 | *CG7442* | 0.79 |
|  |  |  |  |  | BB160009A20D05 | * | *** | 0.87 |
|  |  |  |  |  | BB160009B10B09 | GB11417 | *sim* | 0.80 |
|  |  |  |  |  | BB160009B20B03 | * | *** | 0.85 |
|  |  |  |  |  | BB160012B10D04 | GB16223 | *bif* | 0.89 |
|  |  |  |  |  | BB160013A10F03 | GB19750 | *** | 0.89 |
|  |  |  |  |  | BB160013B10H01 | GB11509 | *futsch* | 0.86 |
|  |  |  |  |  | BB160014A20H04 | GB19097 | *CG32560* | 0.89 |
|  |  |  |  |  | BB160015A10E02 | * | *** | 0.94 |
|  |  |  |  |  | BB160016B20F02 | GB12774 | *CG17149* | 0.85 |
|  |  |  |  |  | BB160019A20F12 | GB18159 | *otk* | 0.74 |
|  |  |  |  |  | BB160020A20A11 | * | *** | 0.78 |
|  |  |  |  |  | BB160022A20G06 | GB18056 | *CG5001* | 0.49 |
|  |  |  |  |  | BB160023A10B09 | GB13144 | *ric8a* | 0.68 |
|  |  |  |  |  | BB160024A20E01 | GB10397 | *** | 0.31 |
|  |  |  |  |  | BB170002A10C02 | GB19995 | *** | 0.28 |
|  |  |  |  |  | BB170002A20F07 | * | *** | 0.88 |
|  |  |  |  |  | BB170002B20H02 | GB14290 | *Strn*Mlck* | 0.90 |
|  |  |  |  |  | BB170011B20H12 | * | *** | 0.92 |
|  |  |  |  |  | BB170014A20C06 | * | *** | 0.87 |
|  |  |  |  |  | BB170016B10B05 | GB17724 | *** | 0.56 |
|  |  |  |  |  | BB170020A20E06 | GB19503 | *** | 0.35 |
