# Supplementary material for: Differential gene expression of the honey bee Apis mellifera associated with Varroa destructor infection
Source: BMC Genomics. 2008 Jun 25;9:301. doi: 10.1186/1471-2164-9-301 (PMC2447852; doi:10.1186/1471-2164-9-301)
Supplement: Additional file 2 — Enrichment analysis on genes that show significant differences in bees parasitized by Varroa (A) and between two different bee genotypes (tolerant and sensitive to Varroa) (B). GO ID and GO term refer respectively to Gene Ontology ID and the associated term. [file 1471-2164-9-301-S2.pdf]

Additional file 2 – Enrichment analysis on genes that show significant differences in bees parasitized by Varroa (A) and between two different bee genotypes (tolerant and sensitive to Varroa) (B).

GO ID and GO term refer respectively to Gene Ontology ID and the associated term.

Biological process								
(A) Varroa					(B) Bee phenotype			
**GO ID**	**GO term**	***P*-value**	**Fly orthologs**		**GO ID**	**GO term**	***P*-value**	**Fly orthologs**
GO:0044267	cellular protein metabolism	0.0017295	*PUf68, Nedd8, CG9520, CG8974, baz, RpS23, sgl, dlg1, CG11859, Pcmt, Stm-Mlck*		GO:0000910	cytokinesis	0.002387	*Mhcl, fwd, Strn-Mlck, kek1*
GO:0007049	cell cycle	0.0025157	*PUf68, baz, dlg1, Dlic2, Strn-Mlck*		GO:0007610	behavior	0.0084653	*sim, Mhcl, para, Dscam, Dhc64C, rogdi, smi21F, bif, otk*
GO:0042053	regulation of dopamine metabolism	0.0084255	*ple*		GO:0007399	nervous system development	0.0142857	*sim, Dscam, gro, fng, Dhc64C, bif, futsch, otk, kek1*
GO:0008333	endosome to lysosome transpor	0.0084255	*Rab7*		GO:0009966	regulation of signal transduction	0.0151664	*gro, fng, kek1, CG32560*
GO:0009792	embryonic development (sensu Metazoa)	0.013913	*baz, sgl, dlg1*		GO:0040011	locomotion	0.0171995	*sim, Mhcl, Dscam, Dhc64C, bif, otk*
GO:0007428	primary tracheal branching (sensu Insecta)	0.0248784	*sgl*		GO:0030091	protein repair	0.0252765	*Pcmt*
GO:0008543	fibroblast growth factor receptor signaling pathway	0.0248784	*sgl*		GO:0046684	response to pyrethroid	0.0252765	*para*
GO:0000003	reproduction	0.0249757	*PUf68, ple, baz, dlg1*		GO:0007175	negative regulation of epidermal growth factor receptor activity	0.0252765	*kek1*
GO:0008285	negative regulation of cell proliferation	0.0485846	*dlg1*		GO:0050771	negative regulation of axonogenesis	0.0252765	*bif*
					GO:0035002	tracheal liquid clearance	0.0252765	*Hr78*
					GO:0046680	response to DDT	0.0252765	*para*
					GO:0006351	transcription, DNA-dependent	0.0260891	*sim, CG11063, CG1815, Alh, gro, RpIII128,*

								Hr78, sirt4, scrt
					GO:0042048	olfactory behavior	0.0423888	*rogdi, smi21F*
					GO:0008590	regulation of frizzled signaling pathway	0.0493011	*gro*
					GO:0045747	positive regulation of Notch signaling pathway	0.0493011	*fng*
**Molecular function**								
**(A) Varroa**					**(B) Bee phenotype**			
**GO ID**	**GO term**	***P*-value**	**Fly orthologs**		**GO ID**	**GO term**	***P*-value**	**Fly orthologs**
GO:0005198	structural molecule activity	0.0032287	*baz, RpS23, dlg1, Dlic2, Strn-Mlck*		GO:0019199	transmembrane receptor protein kinase activity	0.0046603	*Strn-Mlck, otk, kek1*
GO:0005200	structural constituent of cytoskeleton	0.0078132	*dlg1, Dlic2, Strn-Mlck*		GO:0001727	lipid kinase activity	0.0124025	*Pi3K59F, fwd*
GO:0016740	transferase activity	0.0114448	*CG9520, dlg1, Pcmt, CG11859, Strn-Mlck*		GO:0008757	S-adenosylmethionine-dependent methyltransferase activity	0.0125556	*Suv4-20,CG4300, Pcmt*
GO:0016301	kinase activity	0.0215833	*dlg1, CG11859, Strn-Mlck*		GO:0003677	DNA binding	0.0175697	*sim, CG1815, Alh, gro, RpIII128, Hr78, Strn-Mlck, scrt, CG3996, CG15440*
					GO:0046966	thyroid hormone receptor binding	0.0261612	*CG11063*
					GO:0005102	receptor binding	0.0328414	*CG11063, CG1815, CG32560, fng, otk*

					GO:0016740	transferase activity	0.0339263	*Pi3K59F, Suv4-20,CG4300, fwd, Pgk, RpIII128, Strn-Mlck, fng, otk, kek1, Pcmt*
